# Evaluation of the effect of managing oxycodone/acetaminophen as a psychotropic medicine: An interrupted time-series study

**DOI:** 10.3389/fphar.2023.1079972

**Published:** 2023-03-16

**Authors:** Cheng Xiang, Sha Li, Jinwei Zhang, Jieqiong Zhang, Shuchen Hu, Mengyuan Pan, Caijun Yang, Kanghuai Zhang

**Affiliations:** ^1^ Department of Pharmacy Administration and Clinical Pharmacy, School of Pharmacy, Xi’an Jiaotong University, Xi’an, China; ^2^ Center for Drug Safety and Policy Research, Xi’an Jiaotong University, Xi’an, China; ^3^ The Second Affiliated Hospital, Xi’an Jiaotong University, Xi’an, China

**Keywords:** Oxycodone/acetaminophen, policy, clinical effect, psychotropic medicine, interrupted time series

## Abstract

**Background:** Oxycodone/acetaminophen has been reported for misuse for many times in China. To cope with that, Chinese national authorities jointly issued a policy, requiring that oxycodone/acetaminophen should be managed as a psychotropic medicine starting 1 September 2019. This paper aimed to evaluate the effect of this policy in medical institutions.

**Methods:** We used interrupted time-series analysis to examine the immediate level and slope changes in the mean number of tablets prescribed, proportion of oxycodone/acetaminophen prescription exceeding 30 pills, days supplied per prescription and the proportion of days supplied exceeding 10 days with prescription data from 5 tertiary hospitals in Xi’an city between 1 January 2018 and 30 June 2021 (42 months). We divided the prescriptions into two groups, one for long-term drug users, and the other for short-term drug users.

**Results:** In total, 12,491 prescriptions were included in the final study, with 8,941 and 3,550 prescriptions for the short-term and long-term drug users, respectively. Significant differences in the proportion of prescriptions issued by various departments, were observed between pre- and post-implementation of the policy for both short-term and long-term drug users (*p* < 0.001). For short-term drug users, the policy implementation was only associated with an immediate level decrease in proportion of prescriptions exceeding 30 tablets (−4.09%, *p* < 0.001). For long-term drug users, after the policy, the mean number of tablets prescribed and the mean proportion of prescriptions exceeding 30 tablets experienced a level decrease of 22.96 tablets (*p* < 0.001) and a level decrease of 41.13% (*p* < 0.001), respectively; the mean number of days supplied showed a significant level decrease (6.88 days per prescription) and slope increase (0.19 days per month), and the mean proportion of days supplied exceeding 10 days showed a significant level decrease (−10.51% per prescription) and a slope increase (0.27% per month).

**Conclusion:** Implementation of stricter management for oxycodone/acetaminophen achieved its goal of reducing the risk of misuse in short-term drug users. For those long-term drug users, policy needed to be strengthened as the prescription exceeding 10 days was still at a high level after the intervention. Policies targeting patients with different drug demands are needed. Many other strategies can be implemented, including establishing specific guidelines/principles and conducting training programs.

## Introduction

The use of opioid analgesics is a growing problem all over the world. The consumption varied widely throughout the world, with overuse in some countries like the United States and underuse in some low-and middle-income countries (Berterame et al., 2016). The difference in the management strategy may be one of the reasons for that (Hamunen et al., 2009). In China, opioid analgesics are strictly regulated and its use was far below demand (Xie et al., 2020a; Huang et al., 2020). However, drug misuse with opioid, especially those compound formulations, were still reported in multiple studies (Wang et al., 2008; Zhao et al., 2018; Xie et al., 2020a; Tang and Che, 2020; Shen et al., 2021). One example is the oxycodone/acetaminophen.

Oxycodone/acetaminophen is a compound formulation consisting of acetaminophen and oxycodone (Chinese Association for the Study of Pain Committee, 2018). It entered the Chinese market in 1998 and was managed as psychotropic medicine of Category II in the initial stage (Ma et al., 2012). In 2004, it was adjusted to be common medicine (National Medical Products Administration, 2004). Since the management policy of this medicine was relaxed, misuse of that has been reported for many times. In 2010, misuse of oxycodone/acetaminophen in hospitals was detected for the first time (Ma et al., 2012). In 2014, a study reported its overselling in retail pharmacies (Li et al., 2018). In addition, the addiction and dependence was also reported in many studies in the past 5 years (Hu and Cao, 2018; Zhao et al., 2018; Xu et al., 2019; Tang and Che, 2020). Studies on the analysis of rational use of oxycodone/acetaminophen have also found that it was in excessive use in medical institutions (Niu and Zhang, 2017; Geng et al., 2021). The main reason for that was the big difference in the management of common medicines and psychotropic medicines of Category II in China. All the drugs are divided into common drugs and special managed drugs for management in China (Man et al., 2017). The special managed drugs mainly included anesthetic drugs, psychotropic drugs, toxic drugs, radioactive drugs, etc. The psychotropic drugs were further refined and divided into Category I and II. Special managed drugs were subject to a stricter regulation, and supervised by the China National Medical Products Administration, who implemented whole-chain supervision and monitored the usage regularly. To be specific, the differences in the management of common drugs and psychotropic medicines of Category II included production, selling and use (National Health Commission of the People’s Republic of China, 2005; National Health Commission of the People’s Republic of China, 2007). In terms of production, psychotropic medicines of Category II are produced by designated enterprises, while common medicines are produced in enterprises which have the corresponding producing qualifications. In addition to the business license, the institutions selling psychotropic medicines of Category II need to get a specific qualification authentication. In term of use, these two kinds of medicines are also quite different. For common medicine, the prescription duration limit was generally 7 days but it can be appropriately extended to 3 months, while psychotropic medicine of Category II is strictly limited to 7 days. If the prescription duration need to exceed the time limit, the doctor needs to state it clearly and provides an extra signature on the prescription, and the total duration should not exceed 15 days. In addition to the risk of misuse posed by the oxycodone, there are also risks of hepatotoxicity and nephrotoxicity because of the acetaminophen. Nowadays, many studies reported that improper use of acetaminophen, including excessive use and combining with other drugs inappropriately, would cause serious damages, including liver toxicity kidney toxicity and even death (Hiragi et al., 2018; Hopkins et al., 2018; Janković, 2022). In order to avoid the risks of that, the Pharmacopoeia, revised in 2015, clearly stated that the prescription duration of acetaminophen should not exceed 10 days.

In order to cope with the growing misuse of oxycodone/acetaminophen and reduce the risk of that, three Chinese national authorities, including Food and Drug Administration, the Ministry of Public Security, and the National Health Commission, jointly issued a document, requiring that the oxycodone/acetaminophen should be managed as a psychotropic medicine starting 1 September 2019 (National Medical Products Administration, 2019a). This means that stricter restrictions would be applied on the use of oxycodone/acetaminophen.

Until now, several researchers have evaluated the effect of strengthening the management of oxycodone/acetaminophen in medical institutions. An investigation found that the single dose, daily dose, and prescription volume of oxycodone/acetaminophen were all significantly lower after the implementation of the stricter management policy (Tian et al., 2021). Another report also came to similar conclusions (Liu and Wang, 2021). However, all the above studies were conducted in single medical institution and used before-and-after comparative analysis (covering a few months) to describe the short term changes. To comprehensively evaluate the policy effect and obtain reliable conclusion, we conducted a multicenter prescription-based study, quantitatively evaluated the effect of policy and analyzed both the short-term and long-term effects.

## Methods

### Study design and setting

We conducted a retrospective interrupted time-series analysis of oxycodone/acetaminophen prescriptions filled between 1 January 2018 and 30 June 2021 in Xi’an, the capital city of Shaanxi Province.

Shaanxi is a major pilot province of the northwestern health system reform. It has a population of 39.55 million and 11 cities, ranked 12th for gross domestic product *per capita* in Mainland China (China Economic and Social Big Data Research Platform, 2021; Statistics Bureau of Shanxi Province, 2021). In Shaanxi Province, there were 35,300 medical institutions, including 60 tertiary hospitals; most tertiary hospitals were located in its capital city-Xi’an (National Health Commission 2021 of the People's Republic of China, 2021).

### Data source

We invited the top 10 tertiary hospitals in Xi’an in terms of oxycodone/acetaminophen consumption in 2020 to participate the study, and three refused. Among the seven agreed hospitals, two upgraded their Hospital Information System (HIS) in last two years, and could not provide prescriptions during the periods before upgrading. Finally, we extracted the prescriptions data from HIS of five tertiary hospitals. The oxycodone/acetaminophen consumption in these five hospitals accounted for 20% of the total oxycodone/acetaminophen consumption in Shaanxi Province in 2020.

The prescription information included unique prescription code and patients’ code, sex, age of patient, diagnosis, department of physician, drug name and dosage.

### Study cohort and measures

We identified all prescriptions for oxycodone/acetaminophen dispensed between 1 January 2018 and 30 June 2021. Based on the following principal, we excluded the invalid prescriptions and determined the final study cohort: 1) Firstly, we eliminated the prescriptions without key information such as department, age, gender, diagnosis, frequency or dosage; 2) Secondly, the prescriptions prescribed by non-clinical departments were excluded from the study; 3) Thirdly, given that the prescriptions issued by emergency department followed the principle of a 3-day limit, we also excluded prescriptions from that department.

Based on the definition of chronic pain given by WHO (pain which lasts for 3 months or recurs in 3 months), we divided all the drug users into two groups (short-term and long-term drug users) using the information of the total prescription volume, frequency of prescription and the interval between prescriptions. Patients who met one of the following two criteria were considered as long-term drug users: 1) 360 or more tablets (usage covering 3 months) were prescribed during the whole study period; 2) More than one prescription was prescribed between 7 and 90 days and the total number of tablets prescribed was more than 120 tablets, which could be used for pain relief for more than one month. Those who do not met the criteria was regarded short-term drug users. Our analysis was based on the prescriptions prescribed to the short-term and long-term drug users.

The primary outcome for the two groups was mean number of tablets prescribed, which can be used to estimate the consumption of oxycodone/acetaminophen. We also analyzed three secondary outcomes. The first was proportion of oxycodone/acetaminophen prescriptions exceeding 30 pills. The recommended dosage and frequency for oxycodone/acetaminophen is 1 tablet every 6 h (4 pills a day). While according to the policy requirement, one prescription shall not exceed a 7-day supply. In other word, no more than 28 pills would be prescribed for one prescription. And given the facts that there are 10 tablets in a box of oxycodone/acetaminophen and medicines are usually not sold in pieces, we set the standard as 30 tablets. The second indicator was days supplied per prescription, which are calculated based on the pills prescribed and the dosage and frequency. The last indicator was the proportion of days supplied exceeding 10 days which evaluated the relational use of acetaminophen. Acetaminophen is one of the main components of oxycodone/acetaminophen and it is required that the days supplied should not exceed 10 days in the “Instructions for clinical medication in Chinese Pharmacopoeia”.

### Statistical analysis

Firstly, descriptive statistics were used to compare the demographic and characteristics of patients before and after the implementation of the policy. Secondly, to estimate the effect of the policy, we conducted an interrupted time-series analysis of application of oxycodone/acetaminophen. The outcomes and covariates were all aggregated to the level of months. Segmented regression model was used to estimate the changes in the mean number of tablets prescribed, proportion of oxycodone/acetaminophen prescription exceeding 30 pills, mean number of supplied days and proportion of days supplied exceeding 10 days. Two segments with one interruption point were constructed, with 20 months before (January 2018 to August 2019) and 22 months after (September 2019 to June 2021) the intervention. Totally, 42 months were covered in the regression. The model is as follows:

*Y*_*t*_ = *β*_0_+*β*_1_*time_*t*_+*β*_2_*intervention+*β*_3_*time after intervention_*t*_ + *e*_*t*_

*Y*_*t*_ represented the four outcomes. *β*_0_ is the intercept of the outcome variable, estimating the baseline level. *β*_1_ represent the slope of the outcome before the introduction of the policy. *β*_2_ estimates the change in the level of the outcomes immediately after the introduction of the policy. *β*_3_ represents the difference between pre-intervention and post-intervention slopes of the outcome.

The Durbin-Watson and Bgodfrey statistic were used to test for serial autocorrelation. When needed, Feasible Generalized Least Square was used for adjusting for the serial autocorrelation. Breusch-Pagan test was used for heteroscedasticity. In addition, we also tested the normality of the series. All analysis was performed on Stata MP 16.0.

## Results

We extracted 16,133 prescriptions for oxycodone/acetaminophen between 1 January 2018 and 30 June 2021. After exclusion, the final study cohort contained 7,789 drug user, involving 12,491 prescriptions. There were 7,440 short-term and 349 long-term drug users, involving 8,941 and 3,550 prescriptions, respectively. For short-term and long-term drug users, differences in the characteristics were frequent (Table 1). For short-term drug users, the proportion of prescriptions for elderly patients (> 65) increased, while for young patients (≤ 18) decreased after the policy. There is no significant difference in the proportion of prescriptions for male and female before and after the policy for short-term drug users. However, the proportion of prescriptions for short-term and long-term drug users issued by various departments changed greatly. To be specific, the proportion in convenience clinics and surgery dropped, while the proportion prescribed by pain, internal and oncology departments increased.

**TABLE 1:** Demographic and clinical characteristics of the short-term and long-term drug users before and after the policy.

Characteristic			Short-term (*n* = 7,440)				Long-term (*n* = 349)		
			Before (*n* = 3,801)		After (*n* = 3,639)	*p*	Before (*n* = 142)	After (*n* = 207)	*p*
Age	≤18		135 (3.55%)		40 (1.10%)	<0.001	0 (0.00%)	0 (0.00%)	<0.001
	19–64		2,716 (71.45%)		2,462 (67.66%)		70 (49.30%)	111 (53.62%)	
	≥65		950 (24.99%)		1,137 (31.24%)		72 (50.70%)	96 (46.38%)	
Sex	male		2,234 (58.77%)		2,141 (58.83%)	>0.05	87 (61.27%)	106 (51.21%)	<0.001
	female		1,567 (41.23%)		1,498 (41.17%)		55 (38.73%)	101 (48.79%)	
Departments	Surgery		2,288 (60.19%)		1,652 (45.40%)	<0.001	49 (34.51%)	54 (26.09%)	
	Internal medicine		303 (7.97%)		683 (18.77%)		30 (21.13%)	66 (31.88%)	
	Pain medicine		296 (7.79%)		392 (10.77%)		14 (9.86%)	48 (23.19%)	
	Oncology		256 (6.74%)		542 (14.89%)		10 (7.04%)	21 (10.14%)	
	Convenience clinic		233 (6.13%)		38 (1.04%)		29 (20.42%)	3 (1.45%)	<0.001
	Traditional Chinese Medicine		185 (4.87%)		60 (1.65%)		2 (1.41%)	7 (3.38%)	
	Dermatology		141 (3.71%)		41 (1.13%)		1 (0.70%)	0 (0.00%)	
	Infectious Diseases		58 (1.53%)		99 (2.72%)		6 (4.23%)	7 (3.38%)	
	Others		41 (1.08%)		132 (3.63%)		1 (0.70%)	1 (0.48%)	

### Changes in the mean number of tablets prescribed and proportion of oxycodone/acetaminophen exceeding 30 pills

For all users, before the policy, the mean number of tablets prescribed was 22.26 with an upward trend (0.18 per month, *p* = 0.011) and the mean proportion of prescriptions exceeding 30 tablets was 14.35%. After the policy intervention, the mean number of tablets prescribed experienced a level decrease of 3.9 tablets (*p* < 0.01), and the mean proportion of prescriptions exceeding 30 tablets had a significant immediate decrease (-12.24%, *p* < 0.01).

**FIGURE 1:**
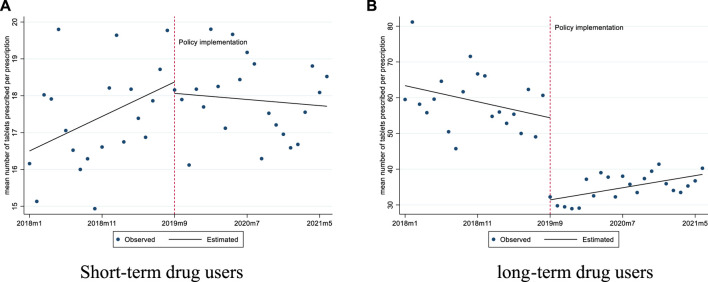
Changes in the mean number of tablets prescribed per prescription for short-term and long-term drug users. **(A)** Short-term drug users **(B)** long-term drug users.

**FIGURE 2:**
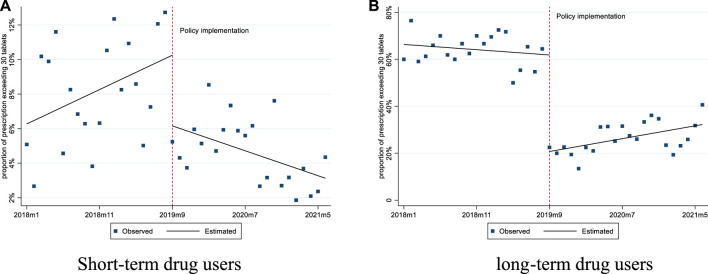
Changes in the proportion of prescription exceeding 30 pills for short-term and long-term drug users. **(A)** Short-term drug users **(B)** long-term drug users.

For short-term drug users, prior to the implementation of the policy, the mean number of tablets prescribed was 16.50 and the mean proportion of prescriptions exceeding 30 tablets was 4.86%. After the policy intervention, the mean proportion of prescriptions exceeding 30 tablets experienced a level decrease of 4.09% (*p* < 0.01), with a downward trend (0.34% per month, *p* = 0.01) (Figures 1, 2; Table 2).

**TABLE 2:** Estimates from Interrupted Time-Series models of the impacts of the policy on the number of tablets prescribed and proportion of prescription exceeding 30 tablets.

		Short-term drug users		Long-term drug users		All users	
		coefficient	*p*-value	coefficient	*p*-value	coefficient	*p*-value
Number of tablets prescribed per prescription							
Intercept	β0	16.50	<0.001***	63.38	<0.001***	22.26	<0.01***
Pre-intervention slope	β1	0.09	0.058*	−0.45	0.172	0.18	0.011**
Level change	β2	−0.31	0.644	−22.96	<0.001***	−3.90	0.001***
Slope change	β3	−0.11	0.062*	0.79	0.024**	0.02	0.797
Proportion of prescription exceeding 30 tablets (%)							
Intercept	β0	6.27%	<0.001***	66.35%	<0.001***	14.35%	<0.01***
Pre-intervention slope	β1	0.20%	0.094*	−0.22%	0.424	0.34%	0.079*
Level change	β2	−4.09%	0.008***	−41.13%	<0.001***	−12.24%	<0.01***
Slope change	β3	−0.34%	0.010**	0.77%	0.028**	−0.02%	0.922

* p < 0.1, **p < 0.05, ***p < 0.01.

For long-term drug users, before the policy, the mean number of tablets prescribed was 63.38, and it experienced a level decrease of 22.96 tablets (*p* < 0.001) after the policy. The mean proportion of prescriptions exceeding 30 tablets was 66.35% before the policy, and we observed a significant decrease of 41.13% in level (*p* < 0.001) after the policy (Figures 1, 2; Table 2).

### Changes in the days supplied and proportion of days supplied exceeding 10 days

For all users, before September 2019, the mean number of days supplied was 8.54, and the mean proportion of days supplied exceeding 10 days was 17.03%. After the policy intervention, the mean number of days supplied showed an immediate level decrease (−1.37 days) and a significant slope increase (0.08 days per month); the mean proportion of days supplied exceeding 10 days decreased significantly by 10.51% immediately (*p* < 0.01), but no significant change in the trend.

**FIGURE 3:**
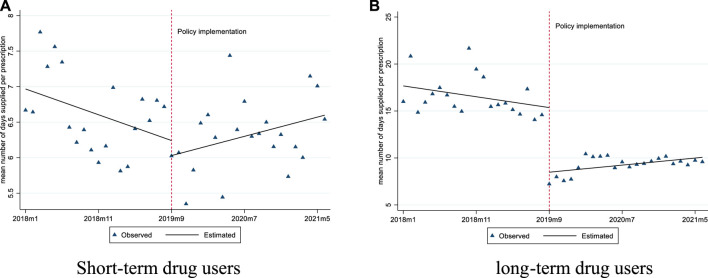
Changes in the mean number of days supplied per prescription for short-term and long-term drug users. **(A)** Short-term drug users **(B)** long-term drug users.

**FIGURE 4:**
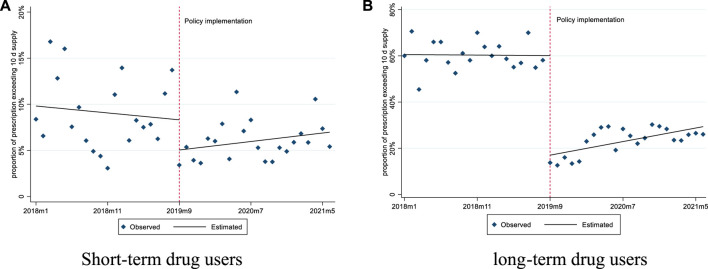
Changes in the proportion of prescription exceeding 10 days’ supply for short-term and long-term drug users. **(A)** Short-term drug users **(B)** long-term drug users.

For short-term drug users, before September 2019, the mean number of days supplied was 6.97 and the mean proportion of days supplied exceeding 10 days was 9.81%. After policy intervention, the mean number of days supplied had a significant slope increase (0.06 days per month), while the mean proportion of days supplied exceeding 10 days did not show any significant changes (Figures 3, 4; Table 3).

**TABLE 3:** Estimates from Interrupted Time-Series models of the impacts of the policy on days supplied and proportion of prescription exceeding 10 days.

		Short-term drug users		Long-term drug users		All users	
		coefficient	*p*-value	coefficient	*p*-value	coefficient	*p*-value
Days supplied per prescription							
Intercept	β0	6.97	<0.001***	17.67	<0.001***	8.54	<0.001***
Pre-intervention slope	β1	−0.04	0.068*	−0.12	0.113	−0.02	0.41
Level change	β2	−0.21	0.468	−6.88	<0.001***	−1.37	<0.001***
Slope change	β3	0.06	0.013**	0.19	0.017**	0.08	0.006***
Proportion of prescription exceeding 10 days(%)							
Intercept	β0	9.81%	<0.001***	60.54%	<0.001***	17.03%	<0.001***
Pre-intervention slope	β1	−0.07%	0.682	−0.02%	0.940	0.11%	0.359
Level change	β2	−3.27%	0.08*	−43.08%	<0.001***	−10.51%	<0.001***
Slope change	β3	0.17%	0.316	0.61%	0.058*	0.27%	0.091*

*p < 0.1, **p < 0.05, ***p < 0.01.

For long-term drug users, before September 2019, the mean number of days supplied was 17.67 and the mean proportion of days supplied exceeding 10 days was 60.54%. After policy intervention, the mean number of days supplied showed a significant level decrease (6.88 days per prescription) and slope increase (0.19 days per month); the mean proportion of days supplied exceeding 10 days also had a significant level decrease (−10.51%) (Figures 3, 4; Table 3).

## Discussion

Previously, some studies have found that strengthening the management significantly reduced the risk of misuse of oxycodone/acetaminophen. The number of prescriptions, dosage, DDDs, drug utilization index, irrational prescription proportion of oxycodone/acetaminophen decreased after the implementation of stricter management (Liu and Wang, 2021; Tian et al., 2021). Our study also confirmed that. In addition, we found the exact effects of the inclusion of oxycodone/acetaminophen in psychotropic drugs and the implementation of stricter regulatory on different group of drug users.

For those short-term drug users, the policy had an impact on the proportion of prescription exceeding 30 tablets, which had a significant level decrease and slope decrease. It also had a level decrease on the proportion of prescription exceeding 10 days (significantly at the level of 0.1). Before the policy, we can find some irrational drug use, as the mean proportion of prescriptions exceeding 30 tablets was 6.27% and the mean proportion of days supplied exceeding 10 days was 9.82%. After the policy implementation, these two indicators decreased. From this aspect, the policy did achieve its anticipated goal of reducing the risks of drug misuse.

For those long-term drug users, all the four indicators decreased immediately. One possibility for the decline was that the longest supply of one prescription was three months before the policy, while after the policy it decreased to 15 days. This has both pros and cons. Long-term drug users have to visit hospitals more frequently which may benefit them as it can help physicians track the progress of the disease, evaluate the efficacy of the treatment, find the adverse effects of drugs and adjust the dosage in time. However, more visits to the hospital for these needful medicines, which increased the cost for patients. More importantly, it caused inconvenience, especially in the time of COVID-19 as cities were locked down frequently.

Besides, we found the proportion of prescription exceeding 10 days supplied among long-term drug users was high, with about 60% and 20% before and after the implementation of the policy, respectively. This should be of high concern, for that the prolonged use of acetaminophen would cause liver and kidney toxicity. One possible reason for this was that oxycodone/acetaminophen was a kind of compound preparation, and doctors was not familiar with it and had no knowledge of the ingredients of that (Chen, 2000; Huang, 2007). Improving the knowledge of doctors about medicine through lectures, training and other ways, was the key to promote the rational use of that.

With the implementation of the policy, the four indicators for long-term drug user increased to a certain extent. The reasons for that are likely multifactorial. Firstly, the administrative supervision of psychotropic drugs of Category II was in certain deficiencies (Xie et al., 2020b). The supervision of “special drugs” focused on anesthetic drugs and psychotropic drugs of Category I, while the supervision of psychotropic drugs of Category II is relatively loose, and corresponding documents on supervision are rare. Therefore, in the initial stage of the policy, medical practitioners may comply with the requirement of the policy as they expected strict regulation. However, if regulation is loose and they think the new policy just caused inconvenience for their patients, practitioners may revert to their old prescription pattern.

Including drugs at risk of misuse in the list of psychotropic drugs, and then adopting stricter management measures are commonly used methods in China to reduce the risk of drug misuse. Until now, three kinds of drugs, including codeine-containing oral liquid preparations, oxycodone/acetaminophen, and remimazolam, have been added to the list of psychotropic drugs due to their increased risk of misuse (National Medical Products Administration, 2015; National Medical Products Administration, 2019a; National Medical Products Administration, 2019b). Our research found that the policy reduced the risk of misuse in short-term drug users but also caused inconvenience for those long-term drug users. Thus, different policies targeting patients with different drug demands are needed. And more importantly, we need promote rational and appropriate use of these medicines by implementing multiple strategies. First of all, it is recommended to further enrich the supporting documents and measures to guide the use of psychotropic drugs and to supervise drug application regulations on the basis of existing laws. Government need further formulate and issue standardized guidelines and principles to guide the clinical use of special drugs in medical institutions. Secondly, the relevant regulatory process and content also need be further developed. Conducting prescription review on such medicine is a good way to ensure its appropriate use in medical institutions. Because prescription review can timely detect relevant problems and avoid improper prescriptions in time. What’s more, training medical practitioners is also needed to provide enough information of how to use it properly and what’s the harm of improper use. Finally, the role of pharmacists should also be brought into full play. Pharmacists are professionals who have better knowledge of drugs and can provide advice about medicines to physicians. Involving pharmacists in the medication process can promote the rational use of drugs and avoid the occurrence of inappropriate drug use to the great extent.

This is the first study to comprehensively evaluate the effects of a policy intervention for including oxycodone/acetaminophen as a psychotropic medicine and the short- and long-term effects are described intuitively. However, our research also has several limitations. Firstly, our data was derived from 5 tertiary medical institutions, not all the medical institutions. However, the consumption in these 5 medical institutions accounted for 20% of all the oxycodone/acetaminophen consumption in Shaanxi, which also makes our sample representative to a certain extent. Secondly, the prescriptions involved in our study was only oxycodone/acetaminophen, and other kinds of opioid analgesics was not involved. In other words, we only evaluated the changes in the prescription of oxycodone/acetaminophen. How many patients shifted to other kinds of opioid analgesics and the changes of prescription pattern of them after policy implementation were not assessed. Thirdly, we divided the patients into long-term and short-term users, but impact of the policy on specific diseases were not included in the analysis.

## Conclusion

This study showed that the policy administrating oxycodone/acetaminophen as a psychotropic medicine achieved its goal of reducing the risk of misuse in short-term drug users. But for those long-term drug users, policies needed to be strengthened as the prescription exceeding 10 days was still at a high level after the intervention. Policies targeting patients with different drug demands are needed. Many other strategies can be implemented, including establishing specific guidelines/principles and conducting training programs.

## Data Availability

The data analyzed in this study is subject to the following licenses/restrictions: Data will be made available on reasonable request. Requests to access these datasets should be directed to JZ, xjtu705010314@163.com.
